# Analyzing the Utility of Openalex to Identify Studies for Systematic Reviews: Methods and a Case Study

**DOI:** 10.1002/cesm.70038

**Published:** 2025-07-24

**Authors:** Claire Stansfield, Hossein Dehdarirad, James Thomas, Silvy Mathew, Alison O'Mara‐Eves

**Affiliations:** ^1^ Evidence for Policy and Practice Information Center, UCL Social Research Institute, Institute of Education University College London London UK

## Abstract

Open access scholarly resources have potential to simplify the literature search process, support more equitable access to research knowledge, and reduce biases from lack of access to relevant literature. OpenAlex is the world's largest open access database of academic research. However, it is not known whether OpenAlex is suitable for comprehensively identifying research for systematic reviews. We present an approach to measure the utility of OpenAlex as part of undertaking a systematic review, and present findings in the context of undertaking a systematic map on the implementation of diabetic eye screening. Procedures were developed to investigate OpenAlex's content coverage and capture, focusing on: (1) availability of relevant research records; (2) retrieval of relevant records from a Boolean search of OpenAlex (3) retrieval of relevant records from combining a PubMed Boolean search with a citations and related‐items search of OpenAlex, and (4) efficient estimation of relevant records not identified elsewhere. The searches were conducted in July 2024 and repeated in March 2025 following removal of certain closed access abstracts from the OpenAlex data set. The original systematic review searches yielded 131 relevant records and 128 (98%) of these are present in OpenAlex. OpenAlex Boolean searches retrieved 126 (96%) of the 131 records, and partial screening yielded two relevant records not previously known to the review team. Retrieval was reduced to 123 (94%) when the searches were repeated in March 2025. However, the volume of records from the OpenAlex Boolean search was considerably greater than assessed for the original systematic map. Combining a Boolean search from PubMed and OpenAlex network graph searches yielded 93% recall. It is feasible and useful to investigate the use of OpenAlex as a key information resource for health topics. This approach can be modified to investigate OpenAlex for other systematic reviews. However, the volume of records obtained from searches is larger than that obtained from conventional sources, something that could be reduced using machine learning. Further investigations are needed, and our approach replicated in other reviews.

## 1 Introduction

Systematic reviews on healthcare topics typically search multiple databases to be as comprehensive as possible, as databases differ in their content and indexing [1]. Translating and running searches between database providers take time, as databases differ on the which fields can be searched, their field names, their search syntax and controlled vocabulary. Time is also needed to combine the search results and remove duplicates. Except for PubMed, many scholarly health research databases are only accessible from commercial database platforms and access varies between systematic review teams. For some, access to commercial platforms is limited or non‐existent, though some versions are available to some low and middle‐income countries by special agreements, such via Research4Life (https://www.research4life.org/).

An advantage of the large commercial platforms is they often have advanced functionalities to support complex Boolean searches, such as wildcards and proximity operators, which are used to both increase sensitivity (recall) and the precision of searches. Furthermore, some health databases have their own controlled vocabulary to aid identification of records rather than only relying on terms used by research authors in the title, abstract and author keywords.

OpenAlex [2] is the world's largest open access database of academic research, containing more than 260 million records across all research disciplines. It incorporates the former Microsoft Academic data set, which ceased publication in 2021, and is updated daily through a multifaceted strategy [3]. If OpenAlex could be used as a single source of references, this could save research time, reduce costs, and facilitate more comprehensive and equitable searching for reviewers with limited commercial database access. However, it is not known whether OpenAlex is suitable for such a task.

The scale and interdisciplinary coverage of OpenAlex provides challenges in aiming for high‐recall comprehensive literature searching, as searches may potentially retrieve many more irrelevant records compared with other resources (apart from searches relying on highly distinctive terminology). While OpenAlex has supported Boolean searching since July 2023, at the time of writing its Boolean search functions are not as sophisticated as those available in PubMed and many commercial platforms. Differences include: (i) there is no detailed controlled vocabulary (though there are 4500 automatically populated topics covering the whole of science [4]); (ii) there are restrictions on how terms are combined with Boolean AND, OR, and NOT; (iii) searches have a lower maximum character limit; (iv) it does not support searching with wildcards (which are used for capturing variations of word forms); (v) it does not use proximity searching between two words, so comparable searches need to be broader (e.g. by combining two words with AND) or more specific (e.g. by searching two words as a phrase). However, OpenAlex uses an ElasticSearch for word forms, so that plural and singular word forms can often be captured without further specification, and searching options continue to evolve [5]. OpenAlex also contains a “network graph,” which means it links records through their citation or textual relations in one of three ways: (i) being referenced in a record (bibliography citations or backward citations); or (ii) being referenced by another record (cited by, or forward citations); or (iii) are ‘related to’ by their textual and source relations through OpenAlex's “related records” algorithm [6].

A recent literature review indicates that OpenAlex is a potentially useful source for identifying literature, though there are no published studies comparing OpenAlex with traditional Boolean literature searches [7]. Hval et al [7] compared coverage in OpenAlex of 860 included studies from three evidence synthesis and found that it contained 97% of studies, though they only located 19% of the 860 studies when they searched for them. However, their methodology was to use connections from the network graph using certain seed records, rather than from Boolean searches (as the Boolean functionality was less developed when their study was undertaken). Rajit et al found OpenAlex coverage of 98.6% of 1249 records used in International Polycystic Ovary Syndrome guidelines [8].

There are two main uncertainties to using OpenAlex as a key source for identifying research for systematic reviews and maps: content coverage and content capture. In terms of content coverage, we do not know how comprehensive OpenAlex is, and if it contains the research records necessary for systematic reviews. Additionally, there is also the possibility that OpenAlex contains important records that are not present in conventional resources. In terms of capturing these records though, we do not know whether (or how) these records can be identified using the search features available in the user interface on the OpenAlex website. Furthermore, there are changes to records that may have implications for capturing content. We have observed modest increases and decreases in the presence of abstract and reference data over time [9], and there have been large‐scale instances of abstract removal from closed access articles owing to the requests of publishers (from Springer Nature, in November 2022, and Elsevier, in November 2024 [10]. An additional uncertainty is whether OpenAlex makes the exclusive use of open access databases a viable proposition. We explored these uncertainties within the context of undertaking a systematic map on the implementation of diabetic eye screening within five countries (Australia, Canada, Ireland, New Zealand and the UK) (described here as “DES map”) [11], and addressed the following research questions:

1. Are records from conventional sources adjudged to be relevant for the map present in OpenAlex? (if not, from where were the remaining records identified?) (**coverage**)
2. Can we find these records through searching OpenAlex using its freely available search functionality? (**capture**)
3. Can we find all the relevant records using one open access bibliographic database and OpenAlex? (**coverage** and **capture**)
4. Does OpenAlex contain records that were not identified from the literature searches for the map? (**coverage**)
  Furthermore, we considered:
5. Can this approach to investigating the utility of OpenAlex be applied to other systematic review contexts?

## 2 Methods

### 2.1 Data Set

The DES map was undertaken using systematic review methods, apart from conducting citation searches, synthesis and critical appraisal. A data set from undertaking the DES map comprised of:

- All the records retrieved from searching multiple databases;
- All the relevant records following full text screening and data extraction;
- All records that were assigned as being eligible based on the title/abstract citation where full‐text could not be retrieved.

The literature search for identifying the records within the DES map was planned as a comprehensive search and included 26 information resources (databases, search engines and website searches) [11]. The main bibliographic database searches (including PubMed) were conducted between 13 and 22 December 2023; supplementary searches using the academic search engines Google Scholar and Bielefeld Academic Search (BASE) were conducted on 24 January 2024; and other online resources and websites were searched during January and February 2024. OpenAlex was searched during July 2024 and the creation date of relevant records in OpenAlex was checked to mitigate the time difference of the original searches for the DES map. The OpenAlex searches were repeated during March 2025 to determine any impact on this study following the removal of closed access abstracts.

## 3 Approach

The methods we used are described here, and Appendix 1 specifies some alternative tools that could be used.

1. **Are records from conventional sources adjudged to be relevant for the map present in OpenAlex?**
  We investigated whether the 131 records of research included in the DES map had equivalent records in OpenAlex using a matching algorithm in EPPI Reviewer [12]. This algorithm automatically matches those records which are highly likely to be the same, and recommends lower confidence matches for manual checking. Records that were not matched were checked in OpenAlex website. This investigation was repeated for the 34 records that were eligible for the map at title and abstract screening but their full‐text was unavailable to the research team (to mitigate potential retrieval bias). Recall was calculated as the percentage of records available in OpenAlex. The records that were not available in OpenAlex were checked to determine how they had been identified for the original map.
2. **Can we find these records through searching OpenAlex using its freely available search functionality?**

i. Boolean searches:
  The EMBASE (OVID) Boolean search strategy used for the DES map was translated into searches of the OpenAlex public website (https://openalex.org/works). Proximity search syntax was replaced with Boolean AND, and word forms other than plurals were specified in place of wildcards. OpenAlex's automatically generated keywords were searched in place of author keywords (topics were not suitably granular in this case). There were also modifications for searching terms relating to country. No limits on publication types were applied. A detailed description of the search strategy translation process is provided in Appendices 2 and 3. Searches were conducted on 12 July 2024 using nine search strings owing to limitations in combining search operators and restricting search string length (refer to these appendices for details). The searches were re‐run on 4 March 2025 to validate the findings.
  Retrieved records were de‐duplicated and records with a publication date before 2003 were removed. The presence of the 131 included records from the DES map were compared with the OpenAlex search results using duplicate functions and manual checks in EPPI Reviewer. Precision and recall were calculated.
ii. Network graph search (citation and related items searches):
  A network graph search (as defined earlier) was executed using the included records that were identified from the OpenAlex Boolean search. This was undertaken for one iteration involving searching OpenAlex via its Application Programming Interface (API) in EPPI Reviewer [13]. (Using EPPI Reviewer was for convenience to undertake this study at scale; the same searches can be carried out using the freely available API and/or using the OpenAlex website using batches of 100 records.) The results were assessed to identify the remaining records that are present in OpenAlex but were not identified from the Boolean searches. Precision and recall were calculated. The searches were repeated on 28 March 2025 using the OpenAlex website to check the feasibility of using this method.
3. **Can we find all the relevant records using one open access bibliographic database and OpenAlex; and how efficiently?**
  The 131 eligible records from the DES map were checked: (i) for their presence in the search results from PubMed from the search conducted on 13 December 2023; and (ii) their presence in OpenAlex. The records meeting these two criteria were used as seed records for a network graph search of OpenAlex, via its API in EPPI Reviewer on 1 August 2024. The results were checked to identify the remaining records that are present in OpenAlex but not identified from the original PubMed search. This was undertaken for one iteration within EPPI Reviewer. Precision and recall were calculated. To aid analysis, the three types of searches were run in separate EPPI Reviewer databases, and in combination (the three types being: bibliography citations, cited by, and related records). The network graph searches were repeated on 24 March 2025 using the OpenAlex website for each of the three types of searches.
4. **Does OpenAlex contain records that were not identified from the literature searches for the map?**
  The results from the OpenAlex searches were de‐duplicated within the original EPPI Reviewer database for the DES map and records published from 2024 onwards were removed. Six machine‐learning classifiers were built and tested within EPPI Reviewer; they used the same underlying algorithm and differed by their training data. The best performing classifier was applied to 17,519 records from the Boolean searches to rank the records by likely relevance. The process of developing and applying the classifier is described in Appendix 4. The highest ranked 2318 records were assessed for eligibility by the same team who screened records for the DES study. This cut‐off was determined from testing and the available time to screen. For newly identified includes, the creation date of the OpenAlex record was checked to determine whether the records would have been available at the time of the original searches or were created afterwards. The best performing classifier was applied to the results from the repeated searches undertaken in March 2025.
5. **Can this approach to investigating the utility of OpenAlex be applied to other systematic review contexts?**
  An internal protocol and working template were drafted a priori, which were reviewed and adjusted during and after implementation. The team reviewed and reflected on the study and developed a new template.

## 4 Results

Appendix 5 presents the flow of literature for the research questions 1, 2, and 4, which involve searches of OpenAlex only.

1. **Are records from conventional sources adjudged to be relevant for the map present in OpenAlex?**
  Of 131 included records, 128 records (97.7%) were present in OpenAlex. Of the remaining three records, one had been identified from searching CINAHL Plus (EBSCO) [14] (an article from the Journal of Diabetes Nursing), and two [15, 16] were from Google Scholar (one a report and one a Masters thesis). Of the 34 records that could not be assessed at full text for the original DES map, nine records (26.4%) were present in the OpenAlex database.
2. **Can we find these records through searching OpenAlex using its freely available search functionality?**
**July 2024 results:**
  The Boolean search in July 2024 yielded a data set of 21,747 records. This contained 126 of the 128 included records known to be present within OpenAlex. We expect the two missed records [17, 18] were not captured by the Boolean searches as they did not contain any details of the country setting in the title and abstract and the authors' country affiliation in the OpenAlex record is blank (the searches used a country restriction, owing to the DES map's focus on five countries) [19, 20, 21]. A network graph search (in July 2024) of the 126 records identified from the original Boolean OpenAlex search yielded 3970 records and identified one of the two missed records [18], which was cited by four of the 126 records. This missed record was originally identified from Boolean searches in CINAHL Plus and from supplementary searches of Google Scholar. The authors of the research are affiliated with healthcare establishments in Gloucestershire, UK and the CINAHL database record contains “United Kingdom” in the Subject field, and “Gloucestershire” in the author affiliation field. The CINAHL search would have captured the record from each of these elements. The other record [17], was not retrieved. It is a preprint within the PsyArXiv on OSF. This OpenAlex record contains no bibliography or cited by data, and the “related items” function links it to 10 records, none of which are about diabetes or eyes from their titles. This record was originally identified for DES map from Google Scholar.
**March 2025 results:**
  The Boolean search in March 2025 yielded 16,573 records and located 123 records (rather than the 126 found in July 2024). The three additional records that were missed in this second search [19, 20, 21] no longer had abstracts in their OpenAlex record. We also note that these records were identified for the DES map from PubMed (and other databases) and their abstracts are still present in their PubMed records. Two records are published in Elsevier's *Canadian Journal of Ophthalmology*, and one published in *Canadian Journal of Public Health* from Springer Science.
  Repeating the network graph search (in March 2025) on the 123 records identified from the Boolean OpenAlex search conducted in March 2025, identified 4276 records (including 112 seed records), and captured four of the five missing records (it included the three records that had originally been identified from the Boolean searches in July 2024, and the one record identified from network graph searches in July 2024).
3. **Can we find all the relevant records using one open access bibliographic database and OpenAlex?**
  Of the 131 records, 117 (89.3%) were identified from PubMed. These 117 records were also identified by the OpenAlex Boolean searches and were used for network graph searches. The PubMed search yielded 2854 records, which corresponds to a precision of 4.1% from the original search. Out of the 14 remaining records, 11 are present on OpenAlex and were all added to OpenAlex by February 2023 (i.e. before any of the searches for the DES map). Five of these records were identified from citation searching of the 117, of which four were in the bibliographies of the seed records and four were records that cited at least one of the 117 seed records. None of the relevant records were found from the related items search. Therefore, six records [17, 22, 23, 24, 25, 26] that were present in OpenAlex were not identified. Five of these six [22, 23, 24, 25, 26] were found from the OpenAlex Boolean search. Table 1 summarizes the recall and precision of the PubMed Boolean search and OpenAlex network graph searches (the latter checked in March 2025).
4. **Does OpenAlex contain records that were not identified from the literature searches for the map?**
  Out of the 2318 OpenAlex records screened, two were records of studies that met eligibility criteria at full‐text and had not been identified for the original DES map [27, 28]. A further three records could not be retrieved at full‐text and another 25 looked relevant though were discovered to be duplicates (or similar, owing to a different author, abstract or journal). The two new eligible records were on OpenAlex at the time the original literature searches for the DES map were undertaken. One record [27] is a report available from the publisher's website (First Nations of Quebec and Labrador Health and Social Services Commission (FNQLHSSC)) and a second [28] is from the publication *International journal of ophthalmic practice*. According to Ulrichsweb (a directory of journals and periodicals), it seems the *International journal of Ophthalmic Practice* is partially indexed in the subscription database British Nursing Index, and we do not have access to this through our institution. Neither of these were identified in the citation and related item searches that were undertaken for research questions 2 and 3. The repeated OpenAlex searches (in March 2025) still identified these two records.

**Table 1:** Recall and precision of a Boolean PubMed search and network graph searches of the included records (*n* = 117) within OpenAlex (March 2025).

Search method		No. of records identified		Recall of relevant records (*N* = 131, of which *n* = 128 are available on OpenAlex)		Precision (% of relevant records as portion of all records identified)	
PubMed Boolean search		2854		117		4.1	
OpenAlex network graph search (bibliography, cited by and related items)	Bibliography (backward citations)	2051	= 4331	4	= 5	0.19	= 0.12
	Cited by (forward citations)	2003		4		0.20	
	Related item	576		0		0	
**Total**		**7185**		**122**		**1.7**

5. **Can this approach to investigating the utility of OpenAlex be applied to other systematic review contexts?**
  Appendix 1 sets out the approach used in this study and alternative open access options. Some process were undertaken in EPPI Reviewer for efficiency purposes (as itis developed in our research center), however, EPPI Reviewer is a not‐for‐profit systematic review tool that requires a subscription after a free monthly trial [29]. The first stage, identifying whether records exist in OpenAlex, could be undertaken using the OpenAlex website rather than from our matching approach. The second stage, Boolean searching of OpenAlex, would be the same, though other teams would need to use openly available options for deduplication and any machine learning. It is possible to undertake network graph searches within the OpenAlex website by searching up to 100 records simultaneously or using the open API with a suitable programming script. Machine learning was only used to determine if new relevant records could be identified. Other options include full manual screening or manual screening on a suitable sample, such as a particular document type (e.g. report) where the volume of results could be lower.

## 5 Discussion

### 5.1 Summary of Key Findings

In terms of OpenAlex content and capture, OpenAlex contained 128 (98%) of the 131 records in the DES map. Boolean searches at the two timepoints yielded at least 94% of the 131 records, with other available records being retrieved by forward and backward citation searches, apart from one record. Two additional relevant records (a journal article and a report) were identified for the DES map. One of these, the report [27], is a notable addition to the DES map as it is only record in the map about training interventions for the referral stage for diabetic eye screening; thus it reduces this study gap in the map.

### 5.2 Discussion of Content and Capture

This ability to identify at least 94% of the original 131 records from Boolean searches seems a remarkably large recall rate considering OpenAlex does not currently facilitate searching on PubMed Medical Subject Headings (MeSH), which are present in the meta‐data and would have made the search more sensitive. Recall is also not affected by the lack of availability of other controlled vocabulary, equivalent to those from commercial databases in this case. However, we have shown that the completeness of records, such as the presence of abstracts or other metadata used in a search (such as country affiliation of authors) will inevitably affect retrieval. This result supports the principle of using OpenAlex as a primary information resource for a health topic, though this finding could differ for searches that rely on controlled vocabulary rather than free‐text terms. Some complex Boolean searches would be more difficult to implement in OpenAlex (such as those containing multi‐stranded nested concepts and search terms in close proximity with each other). However, our capture of two new records from OpenAlex for the DES map shows that OpenAlex can be beneficial to locate research that exists on organizational websites or in journals that are only indexed in subscription databases that may be unavailable to the searcher.

The loss of three records from the OpenAlex searches carried out in March 2025 illustrates the benefit of openly accessible abstracts. Missing records owing to a lack of abstracts is a concern, and there could be potential for systematic bias if research from certain institutions or countries is more likely to be published in closed access abstracts, and alternative resources are not also searched.

Out of the records not identified from the Boolean OpenAlex searches (in July 2024), supplementary searches in Google Scholar and systematic searches in CINAHL provided five records that were either not identifiable or present within OpenAlex [14, 15, 16, 17, 18] (comprising of two journal articles, a preprint, a Masters thesis, and a report) and this supports their use in searches for similar topics if these types of literature are sought. The Google Scholar search, which uniquely provided the preprint, Masters thesis and report, did not aim to be comprehensive. It yielded 2131 records via the Publish or Perish interface through undertaking multiple searches of which the first 500 were saved and collectively de‐duplicated.

### 5.3 Workload Implications

The volume of records obtained from OpenAlex could be a barrier to its use. The Boolean search was generally broader than the original searches, owing the need to AND search terms rather than using proximity searching. Compared to the 12,293 records screened in the actual DES map searches, we retrieved 21,016 records from OpenAlex in July 2024 (and 16,573 in March 2025). We could have manually screened all the records, and machine learning was only used to determine if relevant records of research not identified from the DES map could be obtained from the OpenAlex searches. While we expect that a semi‐automated process using suitable stopping criteria could be feasible based on our approach to address question 4, we did not set out to evaluate this here. Exploring how machine learning could reduce screening workload while maintaining high recall requires further investigation.

Screening volume could also be reduced by using a combination of Boolean searches within PubMed and undertaking network graph searches of relevant records in OpenAlex, with a compromise of recall. This option would have yielded 7185 records to screen and identify 122 of the 131 records (as of March 2025). In this study there was only one iteration of network graph searches, which yielded 4331 in March 2025. This method appears limited by the available citation links. Out of the six available records that the network graph approach did not find (for RQ3), only one has a bibliography, two have been cited once, and four have not been cited. In contrast, the five records that were identified by this method generally had a greater number of records linked by bibliography or from being cited than the six records not found. Related items linkages were not found to be useful in this case. However, we note that related items are not reciprocated across two records (e.g., the related records associated with Sachdeva et al [18] include some of the 117 seed records identified from PubMed searches, though none of these seed records contained this record within their sets of related records.

The yield of the PubMed search was 2854, which is approximately seven times less than the volume of records retrieved from OpenAlex (in July 2024). The PubMed search used proximity functions to reduce the volume of records retrieved (compared with ANDing search terms), and this option is not available in OpenAlex. Therefore, this illustrates that while most of the relevant records may be present in OpenAlex, the current search tools do not enable them to be found as efficiently as other databases. In an additional investigation, which was not part of our planned study, we trained and tested a machine learning classifier on the PubMed search results and applied this to references from the OpenAlex Boolean searches. We observed that full recall of the known relevant would be achieved from screening 3.3% of the OpenAlex search results (630 records), using a screening threshold determined by testing and graphical observation. We are aware this result is dependent on the nature and volume of the training data. However, such an approach shows promise for undertaking rapid searches and where fewer databases are available.

Access to databases is inevitably an issue for most people and institutions (owing to the multitude of databases available), as illustrated by our finding one journal article that may be present in the British Nursing Index (which we did not search). The cost of time spent screening results from an OpenAlex as a core source may be balanced against the cost of subscribing to specific databases. However, the way OpenAlex searches are constructed could be a barrier to some people who typically use subscription databases, similarly in the same way that searchers choose the commercial version of MEDLINE rather than PubMed. In this case, constructing a well‐designed EMBASE (or PubMed search) provided a template to construct the OpenAlex search. In addition, searching PubMed (or other sources) may increase retrieval of records that contain abstracts or metadata that are not in the OpenAlex record. Furthermore, screening the search results of at least one database that provides a high yield of relevant records, could be used to train a classifier to help rank the OpenAlex search results to efficiently boost recall of relevant references. (This requires evaluation of course.).

### 5.4 Replicating This Study

There will be variations in replicating this study. Undertaking searching and using machine classifiers require user interpretation and implementation, and access to database sources for conventional searching varies between institutions and individual reviews. We have also shown there may be variations owing to fluctuations in content of individual records, and network graph relationships are not static.

With regard to undertaking searching, this case study was chosen without considering the feasibility of implementing the search strategy. The case was only chosen because the funder of the DES map permitted a parallel methodological study to be undertaken. However, while it was successfully implemented, some searches may be less feasible than others to undertake in OpenAlex. Adjusting the search to the OpenAlex syntax and running the search in segments requires planning, and the OpenAlex interface and functionality may change in the future. The template database search to translate could be PubMed (or another database), rather than EMBASE. The EMBASE search was used as the template strategy here owing to ease of presentation, and allowed a clearer comparison with the OpenAlex search than was possible with the PubMed strategy. The PubMed search appears unwieldy compared with the EMBASE search owing to the syntax of multiple proximity searches (the DES map search history for PubMed is 39 pages long in a Word document compared with 4 pages for the EMBASE search history).

The machine classifier used in this study was judged to be the most appropriate from testing and did not use all the available training data for the exclude class. This distinction was only possible owing to a hierarchical title and abstract screening which used multiple exclude codes. Other reviews may only contain binary exclude and include data from screening and this could potentially influence the efficiency of the machine learning approach.

### 5.5 Strengths and Limitations

A strength and novelty of this study is demonstrating how a relatively complex Boolean search can be translated into OpenAlex, and some of the benefits and barriers to its use. It also presents an approach to measure the utility of OpenAlex which can be transferred to other contexts. A further strength is that it explores the potential combination of using open access resources for Boolean searching (e.g. PubMed or OpenAlex) combined with citation and related item searching for a mapping review. Testing retrieval against the relatively large volume of gold standard records from the DES map (*N* = 131) increases its utility. Conversely, as it is a map, the quality of the records was not critically appraised. The replication of the OpenAlex searches shows that results can change over time and illustrates caution in drawing conclusions from one case. To draw a clear, generalizable conclusion similar investigations should be undertaken in other reviews. For example, the complexity of searches for healthcare topics vary and for topics with terminology that is more diffuse than used for diabetic retinopathy screening research, there may be challenges in translating this into OpenAlex. Some searches may also benefit from the controlled vocabulary in some conventional databases to a greater extent than shown in this case. It should also be noted that citation relation searches are possible in other databases and results differ between resources [30], and the recent TARCiS statement encourages using two citation indexes to achieve greater coverage of available literature [31].

Time on task was not collected systematically, and the work was undertaken at various intervals over many months. In addition, had the searches been conducted at exactly the same time (rather than the original OpenAlex searches being conducted a few months after the DES map searches), the true volume of results presented from OpenAlex would likely differ, though we do not expect this to affect the results presented from two timepoints of the searches. The overlap between the conventional searches and Boolean OpenAlex has not been fully estimated, and we suspect the overlap of 3,497 records (from the July 2024 searches) is an underestimate owing to the de‐deduplication processes used. Furthermore, we did not screen all the OpenAlex search results, and potentially there may be more unique records that we did not identify. Finally, while a protocol for this study was written in advance, we did not register it.

## 6 Conclusions

Comprehensive literature searching for systematic reviews has always had a tension and bias that trades off the extent of searching with the resources available. OpenAlex appears to be a promising resource for identifying most of the research for a complex but relatively focused search for a health‐related topic and to overcome some barriers of access to research. However, further investigation is needed into machine learning methods for efficiently managing the volume of records from search results. It would be useful to replicate this study on other topics and to evaluate options for streamlining the screening of the search results.

## Author Contributions

**Claire Stansfield:** conceptualization, data curation, formal analysis, investigation, methodology, project administration, validation, writing – original draft, writing – review and editing. **Hossein Dehdarirad:** data curation, investigation, formal analysis, methodology, validation, writing – original draft, writing – review and editing. **James Thomas:** conceptualization, investigation, methodology, writing – original draft, writing – review and editing, formal analysis, supervision. **Silvy Mathew:** data curation, writing – review and editing. **Alison O'Mara‐Eves:** methodology, writing – review and editing, project administration.

## Ethics Statement

This study was conducted following the UK Health Research Authority's research ethics framework. Ethical approval for undertaking the systematic review was granted from the University College London (UCL) Institute of Education Research Ethics Committee (REC1955).

## Conflicts of Interest

The authors declare no conflicts of interest.

## Supporting information

Appendix1‐CES19Dec2024‐updated.

Appendix2 OpenAlex search strategy.

Appendix3 Embase OpenAlex search translation.

Appendix4‐CES19JUNE25 updated.

Appendix5 Literature flows RQ1‐2‐4.

## Data Availability

Data are available from https://osf.io/sndr3/.

## References

[1] C. Lefebvre , J. Glanville , and S. Briscoe , et al., “Chapter 4: Searching for and Selecting Studies,” in Cochrane Handbook for Systematic Reviews of Interventions Version. 6.5.12025, ed. J. P. T. Higgins , T. J. J. Chandler , M. Cumpston , T. Li , M. J. Page , and V. A. Welch . (Cochrane, 2019), cochrane.org/handbook.

[2] J. Priem , H. Piwowar , and R. Orr , OpenAlex: A Fully‐open Index of Scholarly Works, Authors, Venues, Institutions, and Concepts. ArXiv.2022

[3] OpenAlex Support , Where Do Works in OpenAlex Come From? 2024, https://help.openalex.org/hc/en-us/articles/24347019383191-Where-do-works-in-OpenAlex-come-from.

[4] OpenAlex Support , Topics, https://help.openalex.org/hc/en-us/articles/24736129405719-Topics.

[5] OpenAlex Technical Documentation , Search Entities, https://docs.openalex.org/how-to-use-the-api/get-lists-of-entities/search-entities.

[6] OpenAlex technical documentation , Work Object, https://docs.openalex.org/api-entities/works/work-object.

[7] G. Hval , I. Harboe , M. Johansen , M. Larsen , and G. Næss 2023.

[8] D. Rajit , S. McDonald , C. Tay , L. Du , J. Enticott , and H. Teede , “Assessing the Coverage of PubMed, Embase, Openalex and Semantic Scholar for Automated Single Database Searches in Living Guideline Evidence Surveillance: A Case Study of the International PCOS Guidelines 2023,” Journal of Clinical Epidemiology 183 (2025): 111789. 40250535 10.1016/j.jclinepi.2025.111789

[9] H. Dehdarirad and J. Thomas Investigating the Trends in the Abstract and Reference Sections of OpenAlex records. Poster Presented at CORE Information Retrieval Forum; 30 Jan; Newcastle, UK 2025.

[10] B. Kramer , 2024, https://bmkramer.github.io/SesameOpenScience_site/thought/202411_open_abstracts/.

[11] NIHR . Implementation of Screening Guidance/Programmes for Diabetic Eye ‐ NIHR Funding and Awards 2024, https://www.fundingawards.nihr.ac.uk/award/NIHR159996.

[12] J. Thomas , S. Graziosi , and J. Brunton , et al. EPPI‐Reviewer: Advanced Software for Systematic Reviews, Maps and Evidence Synthesis. ed. J. Thomas , S. Graziosi , J. Brunton , Z. Ghouze , P. O'Driscoll , M. Bond and A. Koryakina . (EPPI Centre, UCL Social Research Institute, University College London, 2023).

[13] EPPI Reviewer . OpenAlex in EPPI‐Reviewer, https://eppi.ioe.ac.uk/cms/Default.aspx?tabid=3754.

[14] K. Turner and C. Bodmer , “Improving Retinopathy Screening are we Meeting the NSF Target?,” Journal of Diabetes Nursing 8, no. 9 (2004): 287–290.

[15] C. Hayden, The Barriers and Enablers That Affect Access to Primary and Secondary Eye Care Services Across England, Wales, Scotland and Northern Ireland. RNIB report: RNIB/CEP/IR/01; 2012.

[16] D. B. Kumar , Non‐attendance at Diabetes Eye Services in South Auckland and the Impact of Covid19. [Masters thesis], University of Auckland: researchspace.auckland.ac.nz; 2022.

[17] L. Lewis , R. Povey , J. Taylor , J. Kay , and S. de Souza , “Perceived Barriers and Facilitators to Attending Diabetic Eye Screening Appointments During the COVID‐19 Pandemic,” PsyArXiv (2022), https://osf.io/preprints/psyarxiv/stm3k_v1.

[18] A. Sachdeva , I. Stratton , J. Unwin , R. Moreton , and P. Scanlon Diabetic Retinopathy Screening: Study to Determine risk factors for non‐attendance. 2012.

[19] S. Virani , D. Strong , M. Tennant , et al., “Rationale and Implementation of the Slick Project,” Canadian Journal of Public Health 97, no. 3 (2006): 241–247. 16827417 10.1007/BF03405595 PMC6975836

[20] T. Felfeli , G. Katsnelson , A. Kiss , et al., “Prevalence and Predictors for Being Unscreened for Diabetic Retinopathy: A Population‐Based Study Over a Decade,” Canadian Journal of Ophthalmology 58, no. 4 (2023): 278–286. 35577027 10.1016/j.jcjo.2022.04.002

[21] J. Hwang , C. Rudnisky , S. Bowen , and J. A. Johnson , “Socioeconomic Factors Associated With Visual Impairment and Ophthalmic Care Utilization in Patients With Type Ii Diabetes,” Canadian Journal of Ophthalmology 50, no. 2 (2015): 119–126. 25863851 10.1016/j.jcjo.2014.11.014

[22] K. Shotliff , G. Duncan , R. Dewhirst , et al., “British Association of Retinal Screeners (BARS): Survey of Workload in UK Diabetic Retinopathy Screening Programmes,” Practical Diabetes International 27, no. 4 (2010): 152–154.

[23] K. Xiaoke Li , M. Lovell , K. Evans , and P. H. Gallego , “Reviewing Guidelines on Diabetic Retinopathy Screening in Children and Adolescents With Type 1 Diabetes: Is There Consistency Amongst Practitioners?,” Canadian Journal of Optometry 77, no. 4 (2015): 13.

[24] B. McIntyre , S. Chatterjee , A. Cole , and C. Burren , “Improving the Communication Pathway for Eye Screening in Paediatric Diabetes,” Practical Diabetes 32, no. 3 (2015): 103–106.

[25] I. V. Ho The Role of Tele‐ophthalmology as Part of a Community Health Service to Remote Top End Northern Territory Communities: Cost‐effectiveness Study of Diabetic Retinopathy Screening, Monitoring and Management. 2006.

[26] S. Tanya , B. He , and C. Aubrey‐Bassler , Eye‐Care Utilization Among a Canadian Diabetic Refugee Population: A Retrospective Cohort Pilot Study. Research Square. 2021

[27] J. Briand‐Racine , B. Éthier , É. Grantham , and J. Perreault, Evaluation of the diabetic retinopathy remote screening pilot project among Quebec First Nations. Canada: First Nations of Quebec and Labrador Health and Social Services Commission (FNQLHSSC); 2013 2013.

[28] N. K. Hirji and P. Myers , “Access to a Local Digital Diabetic Retinopathy Screening Service,” International Journal of Ophthalmic Practice 5, no. 5 (2014): 193–196.

[29] EPPI Reviewer , Getting Started. 2024, https://eppi.ioe.ac.uk/cms/Default.aspx?tabid=2914.

[30] M. Gusenbauer , “Beyond Google Scholar, Scopus, and Web of Science: An Evaluation of the Backward and Forward Citation Coverage of 59 Databases' Citation Indices,” Research Synthesis Methods 15, no. 5 (2024): 802–817. 38877607 10.1002/jrsm.1729

[31] J. Hirt , T. Nordhausen , T. Fuerst , H. Ewald , and C. Appenzeller‐Herzog , “Guidance on Terminology, Application, and Reporting of Citation Searching: The TARCIS Statement,” BMJ 385 (2024): e078384. 38724089 10.1136/bmj-2023-078384

