# Supplementary material for: Analyzing the Utility of Openalex to Identify Studies for Systematic Reviews: Methods and a Case Study
Source: Cochrane Evid Synth Methods. 2025 Jul 24;3(4):e70038. doi: 10.1002/cesm.70038 (PMC12302543; doi:10.1002/cesm.70038)
Supplement: Supplementary file 1 — Appendix1‐CES19Dec2024‐updated. [file CESM-3-e70038-s005.docx]

**Appendix 1**

**Steps and procedures for analysing the utility of OpenAlex**

| Step No. | Step | Outline | Process used for this case study | Alternative approaches | Metrics/Reporting |
| --- | --- | --- | --- | --- | --- |
| **1** | **Boolean search of OpenAlex (the searcher is blinded to the eligible records from the original search at this stage)** | Translate search strategy of the main bibliographic database search.  Consider approach used for any simplified searches as a guide for any simplification needed.  Separate searches into sub-searches if necessary. | Search OpenAlex webpage for works (https://openalex.org/works/)  Translate EMBASE search, replacing proximity searches with AND.  (We planned to refer to the Google Scholar search strategy if the EMBASE search was not feasible to translate.) | Not applicable | Volume of records from search (after deduplication of sub-search results) |
| **2** | **Determine which eligible records exist in OpenAlex** | We recommend this step to take place after the step 1 to avoid bias from knowledge of which records exist in OpenAlex. | Apply the OpenAlex matching algorithm to records eligible for the map  Check for matches that are low-confidence or unmatched. | Search and look-up records in OpenAlex webpage for works | % records available in OpenAlex |
| **3** | **Check the presence of the eligible records in the Open Alex search results** | Either search for the known OpenAlex records within the results or deduplicate the known OpenAlex records with the OpenAlex search results. | Check how many of the relevant records are present in the OpenAlex search from duplicate checking. If manual checks are used, re-check the data to reduce errors. | Compare OpenAlex IDs of eligible records present in OpenAlex with those from the search results (from step 3). It is possible to download records from OpenAlex webpage in .csv format and use EPPI Centre's open access file converter to convert the file to RIS (available at <https://eppi.ioe.ac.uk/cms/Default.aspx?tabid=2934>  This RIS file will contain the OpenAlex ID, unlike the RIS file export from OpenAlex. | Precision and recall of OpenAlex search compared with original search results |
| **3** | **Determine which eligible records exist in OpenAlex** | We recommend this step to take place after the sstep 1 to avoid bias from knowledge of which records exist in OpenAlex. | Apply the OpenAlex matching algorithm to records eligible for the map  Check for matches that are low-confidence or unmatched. | Search and look-up records in OpenAlex webpage for works | % records available in OpenAlex |
| 4 | **Network graph searches of OpenAlex** | Run a network graph search on the eligible records that were identified from the OpenAlex Boolean search  To aid analysis, the three types searches were run separately and in combination (the three types being: bibliography citations, cited by, related records). | OpenAlex via its API in EPPI Reviewer. (BringUptoDate)  Run the three different options of searches in separate EPPI-reviews only when analysing the separate contributions of these options | Manual checks in each OpenAlex record in the OpenAlex website.  It is possible to use multiple OpenAlex ID for each citation network graph search (up to 100 IDs, separating each ID with the pipe symbol and no spaces).  For example, the following would search references within the two named OpenAlex IDs:  https://openalex.org/works?filter=cited_by:w4280598166\|w1974257011  Filter notations are:  - Backward citation searches: <https://openalex.org/works?filter=cited_by>:  - Forward citation searches : <https://openalex.org/works?filter=cites>:  - Related records searches: <https://openalex.org/works?filter=related_to>: | Precision and recall  of records available in OpenAlex |
| **5** | **Estimate if OpenAlex contains records that were not identified from the original literature searches** | i) Build, test and apply a machine Learning classifier.  ii) Determine a suitable threshold for stopping screening.  iii) Screen using equivalent methods for original review  iv) Check creation date of the record in OpenAlex | EPPI-Reviewer tools (see description of process in Appendix 2)  For step 5iv), Check created date in the individual record in OpenAlex webpage (click on API and the created date is at the end of the page) | i-iii) use open tools for machine learning  iv) OpenAlex webpage | Methods of building, testing and applying classifier, stopping thresholds,  Volume of records screened, recall |
| **6** | **Check open access bibliographic database (PubMed) and run network graph search in OpenAlex (step 4), based on the eligible PubMed records** | Isolate the full set of records from the PubMed search (before de-duplication) that are the eligible records | EPPI-Reviewer was used to store and screen references, and were labelled by the database source they were identified from.  Manual checks were made within the duplicate records to determine which of the included records in the DES map were retrieved by PubMed. | Any review or information management tool for managing references could be used. The records from the PubMed searches need to be clearly labelled and checked for the presence of eligible records. | Precision and recall of PubMed search compared with original search results , and precision and recall  of additional eligible records available in OpenAlex |
| **7** | **Consider records that are eligible and full-texts are not retrievable, and how many of these are on OpenAlex** | Check and report how many of the records that could be relevant and not retrievable at full text in the review, and therefore not possible to determine eligibility. Check how many of these records are on OpenAlex, | EPPI-Reviewer was used to record data about the full-text retrieval status of the eligible records. The presence on OpenAlex was checked through the matching algorithm and manual checks on the OpenAlex webpage. | Any review or information management tool or spreadsheet could be used for documenting which of the eligible records were not retrieved at full-text.  Search and look-up these records in the OpenAlex webpage for works | Volume of records where full text is not retrievable. |
