# Supplementary material for: Analyzing the Utility of Openalex to Identify Studies for Systematic Reviews: Methods and a Case Study
Source: Cochrane Evid Synth Methods. 2025 Jul 24;3(4):e70038. doi: 10.1002/cesm.70038 (PMC12302543; doi:10.1002/cesm.70038)
Supplement: Supplementary file 2 — Appendix2 OpenAlex search strategy. [file CESM-3-e70038-s004.docx]

**Appendix 2: OpenAlex search strategy**

The EMBASE search was translated into nine search strings which were separately run using the OpenAlex website on 12 July 2024. The search strings were re-run on 4 March 2025 due to changes in the availability of abstracts within some OpenAlex records. The number of retrieved records of the latest searches presented in parentheses.

Searches #1-6 are the same for the 'diabetic eye screening' concept, and differ in terms of country concept terms and filter attributes. Search line 1 examined country names and adjectives within full-text records. Searches #2-5 utilised the country terms from the EMBASE search in the title and abstract. Owing to search character limitations four search strategies were undertaken. Search #6 focused on the country of author affiliation.

Searches #7 and #8 used the keywords filter attribute that searches keywords that are automatically assigned to each record. This was to overcome the limitation of OpenAlex's absence of author keyword search capabilities. Country terms were not included for these search lines, given the relatively small number of records retrieved by the keyword search.

Search #9 targeted diabetic eye screening program names within the countries of interest.

The retrieved results of all search lines were exported in RIS file format from the OpenAlex website and 45,696 records were imported into EPPI-Reviewer. After removing duplicate records and applying a publication year limit of 2003 onwards, 21,747 records considered for further analysis. Total number of retrieved records on 4 March 2025 was 30,781. After removing duplicate records and limiting the records to those published 2003 onwards, 16,573 records remained for additional analysis. We note that it is also possible to restrict by publication year in the searches on the OpenAlex webpage.

**Key:**

| **Notation** | **Explanation** |
| --- | --- |
| \| | Pipe symbol used for Boolean 'OR'  (Boolean AND and NOT were not used in this case, but would use + and ! symbols respectively) |
| , | Comma symbole used for ANDing between filters |
| title_and_abstract.search: | Title and abstract filter |
| keywords.id:keywords/ | Keyword filter |
| authorships.countries:countries/ | Country affiliation of author filter |
| publication_year:2003-2024 | Publication_year dated between 2003 and 2024 |

**Search strings**

#1

https://openalex.org/works?filter=title_and_abstract.search:Diabetic|diabetics|diabetes,title_and_abstract.search:Eye|eyes|retina|retinal|retinopathy|macula|macular|maculopathy|vision,title_and_abstract.search:screen|screening|diagnosis|diagnoses|detection|test|tests|testing|program|programs|programme|programmes|care|exam|exams|examination|examinations|checks|check-up|check-ups|checkup|checkups|surveillance|monitor|monitoring|teleophthalmology|"tele-ophthalmology"|teleretina|teleretinal|teleretinopathy|"tele-retina"|"tele-retinal"|"tele-retinopathy"|"RetinaScreen"|SLICK|"keep sight",default.search:UK|"United Kingdom"|"u. k."|"Britain"|"British"|England|Wales|Welsh|Scotland|Scottish|Ireland|Irish|Canada|Canadian|canadien|Australia|Australian|"New Zealand"|"New Zealander"

Number of results: 11,106 (7,851)

#2

https://openalex.org/works?filter=title_and_abstract.search:Diabetic|diabetics|diabetes,title_and_abstract.search:Eye|eyes|retina|retinal|retinopathy|macula|macular|maculopathy|vision,title_and_abstract.search:screen|screening|diagnosis|diagnoses|detection|test|tests|testing|program|programs|programme|programmes|care|exam|exams|examination|examinations|checks|check-up|check-ups|checkup|checkups|surveillance|monitor|monitoring|teleophthalmology|"tele-ophthalmology"|teleretina|teleretinal|teleretinopathy|"tele-retina"|"tele-retinal"|"tele-retinopathy"|"RetinaScreen"|SLICK|"keep sight",title_and_abstract.search:"United Kingdom"|UK|Britain|British|england|"u. k."|"Isle of Man"|"Channel Islands"|Guernsey|Hebrides|London|Ireland|irish|Scotland|scottish|Wales|Welsh|bath|birmingham|bradford|brighton|bristol|carlisle|cambridge|canterbury|chelmsford|chester|chichester|coventry|derby|durham|ely|exeter|gloucester|hereford|hull|lancaster|leeds|leicester|lincoln|liverpool|manchester|newcastle|norwich|nottingham|oxford|peterborough|plymouth|portsmouth|preston

Number of results: 8,350 (4,535)

#3

https://openalex.org/works?filter=title_and_abstract.search:Diabetic|diabetics|diabetes,title_and_abstract.search:Eye|eyes|retina|retinal|retinopathy|macula|macular|maculopathy|vision,title_and_abstract.search:screen|screening|diagnosis|diagnoses|detection|test|tests|testing|program|programs|programme|programmes|care|exam|exams|examination|examinations|checks|check-up|check-ups|checkup|checkups|surveillance|monitor|monitoring|teleophthalmology|"tele-ophthalmology"|teleretina|teleretinal|teleretinopathy|"tele-retina"|"tele-retinal"|"tele-retinopathy"|"RetinaScreen"|SLICK|"keep sight",title_and_abstract.search:ripon|salford|salisbury|sheffield|southampton|"st albans"|stoke|sunderland|truro|wakefield|wells|westminster|winchester|"wolverhampton"|worcester|york|bangor|cardiff|newport|"st asaph"|"st davids"|swansea|aberdeen|dundee|edinburgh|glasgow|inverness|stirling|armagh|belfast|lisburn|londonderry|derry|newry|ulster|Canada|canadian|canadien|alberta|Manitoba|"New Brunswick"|"Newfoundland and Labrador"|"Northwest Territories"|"Nova Scotia"|Nunavut|Ontario|"Prince Edward Island"|Quebec|Saskatchewan|"Yukon Territory"

Number of results: 14,260 (10,058)

#4

https://openalex.org/works?filter=title_and_abstract.search:Diabetic|diabetics|diabetes,title_and_abstract.search:Eye|eyes|retina|retinal|retinopathy|macula|macular|maculopathy|vision,title_and_abstract.search:screen|screening|diagnosis|diagnoses|detection|test|tests|testing|program|programs|programme|programmes|care|exam|exams|examination|examinations|checks|check-up|check-ups|checkup|checkups|surveillance|monitor|monitoring|teleophthalmology|"tele-ophthalmology"|teleretina|teleretinal|teleretinopathy|"tele-retina"|"tele-retinal"|"tele-retinopathy"|"RetinaScreen"|SLICK|"keep sight",title_and_abstract.search:Vancouver|Victoria|Calgary|Edmonton|Regina|Saskatoon|Winnipeg|Ottawa|"McMaster"|"thunder bay"|Montreal|"McGill"|Moncton|Fredericton|"prince edward island"|charlottetown|"nova scotia"|halifax|Dalhousie|labrador|newfoundland|nunavut|iqaluit|"northwest territor"|yellowknife|whitehorse|"first nation"|metis|inuk|innu|innuit|autochtone|Hamilton|Kingston|Sudbury|Laval|Sherbrooke|Nunavik|Kuujjuaq|Inukjuak|Puvirnituq|australia|"Australian Capital Territory"|"New South Wales"|"Northern Territory"|Queensland|"South Australia"|Tasmania|Victoria|"Western Australia"|"New Zealand"|"NZ"|Otago

Number of results: 2154 (1202)

#5

https://openalex.org/works?filter=title_and_abstract.search:Diabetic|diabetics|diabetes,title_and_abstract.search:Eye|eyes|retina|retinal|retinopathy|macula|macular|maculopathy|vision,title_and_abstract.search:screen|screening|diagnosis|diagnoses|detection|test|tests|testing|program|programs|programme|programmes|care|exam|exams|examination|examinations|checks|check-up|check-ups|checkup|checkups|surveillance|monitor|monitoring|teleophthalmology|"tele-ophthalmology"|teleretina|teleretinal|teleretinopathy|"tele-retina"|"tele-retinal"|"tele-retinopathy"|"RetinaScreen"|SLICK|"keep sight",title_and_abstract.search:Canterbury|Nelson|Wellington|Taranaki|Auckland|"Hawke's bay"|"Hawkes bay"|Aotearoa|Eire|"New Zealander"|"New Zealanders"|maori|polynesian|northland|waitemata|Manukau|waikato|"lakes district"|tairawhiti|aboriginal|"torres strait islander"|Sydney|melbourne|Cork|Dublin|Galway|Waterford|Maynooth|Limerick|Cashel|Kilkenny|Drogheda|Aston|Sussex|Kent|Ulster|Keele|"East Anglia"|Warwick|"St Andrews"|Strathclyde|Cranfield|Essex|"Anglia Ruskin"|Waterloo|"Western University"|Guelph|"Simon Fraser"

Number of results: 1353 (672)

NB: two abbreviations "IRL" and "CAN" were excluded from the search strategy due to a dramatic increase in the number of results.)

#6

https://openalex.org/works?filter=title_and_abstract.search:Diabetic|diabetics|diabetes,title_and_abstract.search:Eye|eyes|retina|retinal|retinopathy|macula|macular|maculopathy|vision,title_and_abstract.search:screen|screening|diagnosis|diagnoses|detection|test|tests|testing|program|programs|programme|programmes|care|exam|exams|examination|examinations|checks|check-up|check-ups|checkup|checkups|surveillance|monitor|monitoring|teleophthalmology|"tele-ophthalmology"|teleretina|teleretinal|teleretinopathy|"tele-retina"|"tele-retinal"|"tele-retinopathy"|"RetinaScreen"|SLICK|"keep sight",authorships.countries:countries/gb|ie|ca|au|nz

Number of results: 7851 (6184)

#7

https://openalex.org/works?filter=keywords.id:keywords/diabetic-retinopathy,keywords.id:keywords/screening|screening-uptake

Number of results: 104 (0)

#8

https://openalex.org/works?filter=keywords.id:keywords/diabetes|diabetes-mellitus|type-1-diabetes|type-2-diabetes|diabetic-complications|diabetes-complications,keywords.id:keywords/retinopathy|maculopathy

Number of results: 204 (0)

#9

https://openalex.org/works?filter=title_and_abstract.search:retinascreen|retinascreening|"DES program"|Keepsight|"Diabetic eye screening programme"|"Diabetic eye screening programmes"|"Diabetic eye screening program"|"Diabetic eye screening programs"|"Irish national diabetic retinascreen"|"teleophthalmology screening program"|"National diabetes eye screening program"|"National diabetes eye screening programs"|"National diabetes eye screening programme"|"National diabetes eye screening programmes"|"Diabetic Retinal Screening"|"Diabetes Eye Care Pathway Toolkit"|"Manitoba Retinal Screening Vision"|"Pan-Ontario diabetic retinopathy screening"|"Ontario Health Teleophthalmology Screening"|"Ontario Health's Teleophthalmology Screening"|"Toronto Teleophthalmology"|"Toronto tele-retina"|"Toronto teleretina"|"Toronto tele retina"|"Screening for Limb"

Number of results: 311 (279)
