# Supplementary material for: Analyzing the Utility of Openalex to Identify Studies for Systematic Reviews: Methods and a Case Study
Source: Cochrane Evid Synth Methods. 2025 Jul 24;3(4):e70038. doi: 10.1002/cesm.70038 (PMC12302543; doi:10.1002/cesm.70038)
Supplement: Supplementary file 4 — Appendix4‐CES19JUNE25 updated. [file CESM-3-e70038-s001.docx]

**Appendix 4**

**Building, Testing and Applying the Classifier for Research Question 4**

*Building and testing the classifier*

Six classifiers were built and tested based on the screening decisions of the literature searches of the DES map. Testing of the classifier involved randomly sampling 25% of full-text includes (n=33) so that they were not included in the training data of the six classifiers so that their relevance rankings could be observed for each classifier. This process was repeated for the two best performing classifiers (with the ideal being a *J-shaped curve* showing a high edge at *the highest* relevance rankings). In addition, a random sample of 5% of the new OpenAlex results (n=912) was used to gauge the performance of relevance ranking of the two best-performing classifiers (with the ideal seen being either a U-shaped curve or a *reverse* J-shaped curve showing a high edge at the *lowest* relevance rankings (the ratio of includes and excludes determines if the curve is U or J-shaped)). Once the best classifier was determined, the classifier was re-built using all the available included records; it was built on 1,251 records that were includes and 5,348 excluded on the criterion of not being about diabetic eye, and both record sets were based on the decisions made at the title and abstract stage. The two test-versions of the best classifier (i.e. with 25% full-text includes removed), showed the lowest-ranking includes had scores of 33 and 40 (on a scale of 0-99), respectively, and this performance was informative for estimating a potential cut-off threshold for applying the re-built classifier.

*About the classifier*

The classifiers used a simple logistic regression on uni-, bi- and tri-gram bag of word features transformed into vectors according to a tf-idf algorithm. The source code used was the same as published here: <https://github.com/EPPI-Centre/CochraneCOVID19Classifier>.

*Applying the classifier*

Figure 1 shows a bar chart of the relevance-ranking scores from applying the final classifier to the 17,519 records from OpenAlex. Those with relevance-ranking scores of 30 and above were screened, which corresponded to 2,318 records. A screening limit was necessary based on the time available to conduct the study.


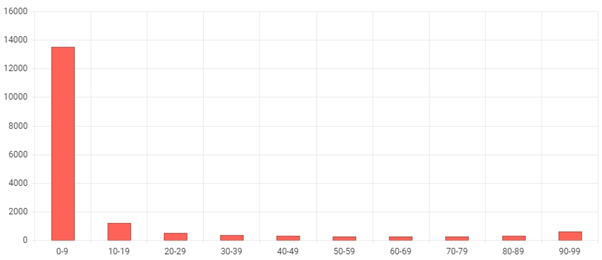


**Figure 1** Relevance scores applied to the records from OpenAlex searches following deduplication from the original searches of the DES map (n=17,519)
