# Supplementary material for: Analyzing the Utility of Openalex to Identify Studies for Systematic Reviews: Methods and a Case Study
Source: Cochrane Evid Synth Methods. 2025 Jul 24;3(4):e70038. doi: 10.1002/cesm.70038 (PMC12302543; doi:10.1002/cesm.70038)
Supplement: Supplementary file 5 — Appendix5 Literature flows RQ1‐2‐4. [file CESM-3-e70038-s002.pptx]

## Slide 1
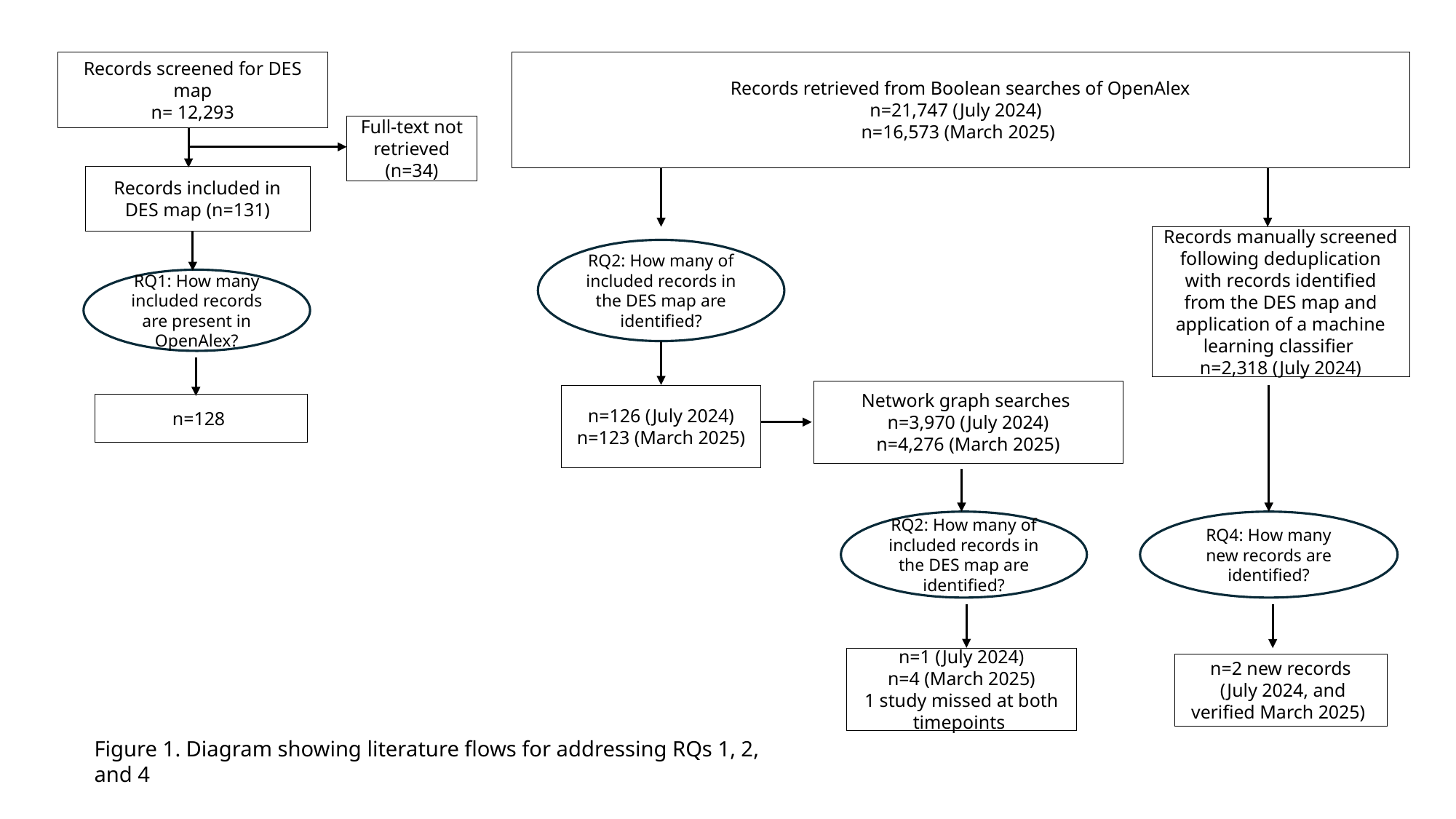

Records screened for DES map
n= 12,293
Records retrieved from Boolean searches of OpenAlex
n=21,747 (July 2024)
n=16,573 (March 2025)
Full-text not retrieved (n=34)
Records included in DES map (n=131)
Records manually screened following deduplication with records identified from the DES map and application of a machine learning classifier
n=2,318 (July 2024)
RQ2: How many of included records in the DES map are identified?
RQ1: How many included records are present in OpenAlex?
Network graph searches
n=3,970 (July 2024)
n=4,276 (March 2025)
n=126 (July 2024)
n=123 (March 2025)
n=128
RQ4: How many new records are identified?
RQ2: How many of included records in the DES map are identified?
n=1 (July 2024)
n=4 (March 2025)
1 study missed at both timepoints
n=2 new records
 (July 2024, and verified March 2025)
Figure 1. Diagram showing literature flows for addressing RQs 1, 2, and 4
